# Benefits of Adding an Aquatic Resistance Interval Training to a Nutritional Education on Body Composition, Body Image Perception and Adherence to the Mediterranean Diet in Older Women

**DOI:** 10.3390/nu13082712

**Published:** 2021-08-06

**Authors:** Alejandro Martínez-Rodríguez, Bernardo J. Cuestas-Calero, María Martínez-Olcina, Pablo Jorge Marcos-Pardo

**Affiliations:** 1 Department of Analytical Chemistry, Nutrition and Food Science, Faculty of Sciences, University of Alicante, 03690 Alicante, Spain; maria.martinezolcina@ua.es; 2 Alicante Institute for Health and Biomedical Research (ISABIAL Foundation), 03010 Alicante, Spain; 3 Faculty of Sport, San Antonio Catholic University of Murcia, 30107 Murcia, Spain; bjcuestas@alu.ucam.edu; 4 Department of Education, Faculty of Education Sciences, University of Almería, 04120 Almería, Spain; pjmarcos@ual.es; 5 SPORT Research Group (CTS-1024), CERNEP Research Center, University of Almería, 04120 Almería, Spain

**Keywords:** geriatric rehabilitation, aging, nutrition education, aquatic resistance training

## Abstract

The human population is increasing due to lengthening life expectancy, but the quality of life and health of people is moving in the opposite direction. The purpose of this study is to evaluate how aquatic resistance interval training can influence body composition, body image perception and adherence to the Mediterranean diet (MD) in older women participants in a nutrition education program and to study the relation between these variables. Thirty-four participants aged 69 ± 4 years were randomly assigned into two groups: experimental (aquatic resistance interval training plus nutritional intervention) and control (nutritional intervention). The intervention consisted of resistance training in an aquatic environment carried out for 14 weeks (three sessions per week; 60 min each). Body composition, body image perception and adherence to MD diet were evaluated at baseline and 14 weeks. No significant differences were found between groups regarding body image perception and adherence to the MD. There was a significant increase in muscle mass (kg) (*p* < 0.001) and a significant decrease in fat mass (kg) (*p* < 0.001) in the intervention group when compared to the control group. The addition of aquatic resistance interval training to a nutritional intervention was not sufficient to change body image perception and adherence to MD but produced improvement in body composition (through an increase in muscle mass and decrease on fat mass) in older women.

## 1. Introduction

Aging is characterized by a progressive decline in muscle strength, which potentially impacts mobility and translates into frailty and functional disability, especially in the lower extremities [1].

Around the age of 50 years, women reach menopause. Menopause is characterized by hormonal changes that include a decline in estrogen level, which has an important role in bone remodeling [2], cardiovascular disease and mortality [3] in females. Some researchers explain that the absence of estrogen may be a relevant triggering factor for obesity [4]. Estrogen deficiency enhances metabolic dysfunction, predisposing the human body to diabetes mellitus type 2, metabolic syndrome and cardiovascular disease [4]. 

The perimenopausal phase—the time in which a woman transitions to menopause—centers around shifts to the hormonal system, which are associated with a weight gain, an increase in fat mass [5] and a reallocation of body fat from the lower body (i.e., hips) to the upper body (i.e., waist and torso) [6]. Given that these shifts are in direct contrast to Western society’s young, thin, beauty standard, menopause could be an especially critical window of vulnerability for the development or exacerbation of disordered eating behaviors and attitudes, highlighting body shape image disorders.

Furthermore, there is a direct relation between loss of bone mass and microarchitectural deterioration of bone tissue, and a decrease in bone strength added to subsequently increased fracture risk, which eventually leads to conditions clinically known as osteopenia and osteoporosis [7], which are major health problems. A mechanical stimulus is then needed in order to maintain bone health.

Many parts of the brain that are related to aging may also be sensitive to shifts in hormone levels: for example, gonadal changes, which usually occur around mid-life, are thought to be associated with changes in cognitive function [8], and mood symptoms are well known to be habitual during the menopause transition period [9,10]. Mood symptoms such as depression and anxiety, along with hot flushes and night sweats, may be affiliated with a negative experience of menopause [8]. The experience of menopause is influenced by the cultural and social context. Women who live with a chronic mental health state can experience additional or increased symptoms throughout menopause [8]. 

In addition, it is important to consider the eating habits of this population. Some research suggests that postmenopausal women present a greater eating disinhibition and dietary restraint compared to premenopausal women [11]. It has been suggested that higher adherence to a healthy dietary pattern, such as the Mediterranean diet (MD), is contrarily associated with being overweight/obese in perimenopausal and postmenopausal women. High adherence to the Mediterranean dietary pattern and a body mass index (BMI) of 25 kg/m^2^ or lower might make a women’s quality of life better in the postmenopausal phase [12].

The traditional Mediterranean dietary pattern is distinguished by abundant consumption olive oil (the major source of fat), plant foods (vegetables, fruits, cereals and nuts), fresh fruit as a daily dessert, low to moderate intake of dairy products (cheese and yogurt), low intake of red meat, low to moderate intake of fish and poultry and regular moderate intake of wine, generally consumed during meals.

Regular physical activity (PA) is considered an important element of lifestyle. Numerous epidemiological studies have proved that it has a positive influence on reducing the incidence of many diseases and mortality [13,14,15]. Regular PA also helps to preserve functional abilities, which play a vital role in motor resourcefulness and self-reliance in everyday life, contributing to a better quality of life and positive self-esteem [16]. In older adults, the best prevention for the accelerated decline in muscle strength and mass is performing resistance training [1], which has been a fundamental part of the American College of Sports Medicine (ACSM) exercise prescription guidelines for older adults since 1998 [17,18]. Safely applied, resistance training has been shown to improve lower and upper body muscle strength in the older adult population, including those suffering from comorbidities such as stroke, postmortem, coronary bypass, hypertension and obesity [1]. 

Exercise in water, often referred to as water-based exercise, presents a lower risk of traumatic fracture; moreover, the joints are exposed to less stress and impact (reduced loading due to buoyancy) compared to land-based exercise such as running, strength training and resistance training [7]. Furthermore, water-based exercise has been highly recommended for older people, especially those with disability, due to the reduced pain and increased security it can provide, as well as the additional benefits for neuromuscular/functional fitness and cardiometabolic health.

Therefore, menopause marks a period in a woman’s life where it is relevant to introduce preventive strategies to reduce the risk of suffering cardiovascular disease, bone health and mortality [2,3].

The main objective of the current study was to evaluate the effect of the addition of aquatic resistance interval training to a nutritional intervention on body composition, body image perception and adherence to MD in older women.

## 2. Materials and Methods

### 2.1. Study Design 

This study was a randomized clinical trial in which the participants were allocated to an experimental group (aquatic resistance interval training) plus nutritional intervention and a control group (nutritional intervention) in order to determine the effectiveness of aquatic resistance interval training on the variables of body composition, body shape and adherence to the MD. The subjects were assigned electronically in a random way by block design into two arms (control and experimental) using online computer software as stated by published recommendations [19]. This procedure was performed by a researcher who was not involved in the interventions or evaluations of this study.

### 2.2. Participants

This study included only female older adults. Forty-five women over the age of 65 years from Alicante, Spain, participated. The inclusion criteria were: to be over 65 years old; not to have undergone surgery in the last year; not to present musculoskeletal, neurological or orthopedic diseases that could affect the ability to perform the tests; to be able to walk independently without orthopedic assistance; and not to have previously performed any of the tests included in the study.

**Figure 1:**
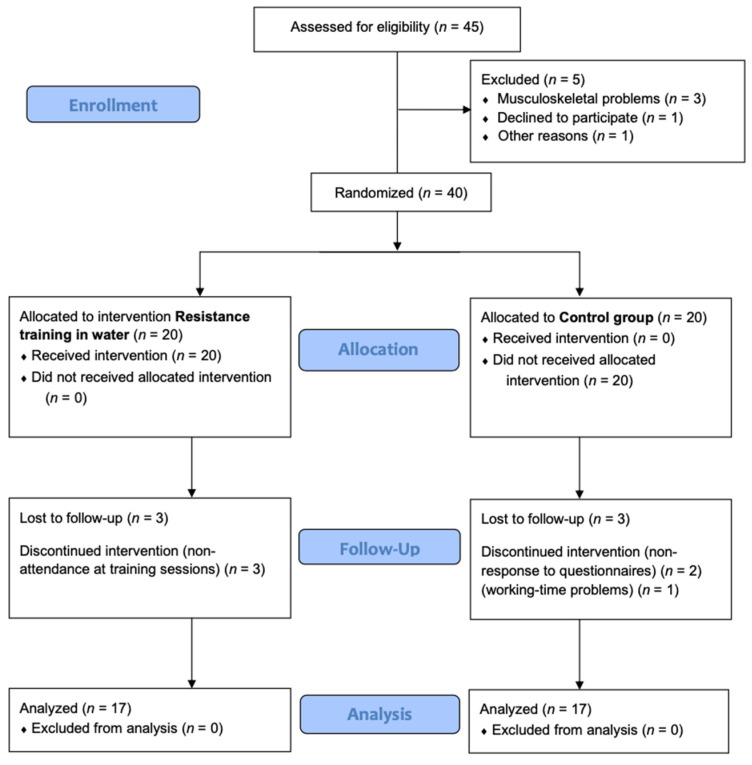
Consort 2010 flow diagram.

Five participants did not meet the inclusion criteria: one declined to participate, and the other four were not able to participate because of musculoskeletal mobility problems, leaving 40 participants who were randomly allocated to an aquatic resistance-training group and a control group. Over the follow-up period, six participants withdrew from the trial, three from each group. Consequently, just 34 women were involved in the analysis. Both groups presented no differences in the demographic variables, and all withdrawals were because of personal reasons (Figure 1).

### 2.3. Declarations: Ethical Approval, Consent to Participate and Consent for Publication

The present study was carried out in agreement with the standards of the Helsinki Declaration. The Human Research Ethics Committee of the Catholic University of Murcia (Spain) gave approval to run a randomized trial (CE061920) and prior to the experiment all study participants provided written consent. Furthermore, researchers kept the participants’ personal data confidential by codifying all personal information.

### 2.4. Study Intervention

Evaluation methods regarding body composition, body image perception and adherence to a Mediterranean diet were administered to both groups at baseline and after intervention (14 weeks). 

#### 2.4.1. Aquatic Resistance Interval Training

The intervention consisted of a training programme in an aquatic environment. Supervised resistance training was performed for 14 weeks. The sessions were conducted in a heated pool three times a week for 60 min per session. The sessions began with a 15 min warm-up consisting of aerobic and resistance exercises (10 min) and stretching (5 min) of all the muscle groups involved, followed by 30 min of comprehensive interval resistance training involving four 5 min sessions with a 2 min rest between each session.

In each session, the same exercises (pectoral/back, hip flexor/extensor, biceps/triceps, knee flexor/extensor, shoulder and core) were performed for 1 min consecutively, with intervals of 30, 20 and 10 s [20] and at low, moderate and high perceived intensity, respectively [21]. According to the perceived exertion scale, when participants needed to increase the intensity of the upper hemisphere exercises, they put on resistance gloves or resistance dumbbells, whereas for the lower-hemisphere exercises, they put on resistance anklets.

Finally, in the last 10–15 min, stretching (5 min) and relaxation exercises (10 min) were performed. In all the intervention sessions, the perception of effort was controlled using the Borg scale [22].

#### 2.4.2. Nutritional Education

Additionally, all participants received the same nutritional education, based on the MD divided into four theoretical and practical workshops of 60 min for 14 weeks in order to provide updated information about the benefits of following an adequate food pattern. Trained dietitians conducted the sessions. The topics covered in the sessions were: (1) food and nutrition, MD pyramid and a modern lifestyle—daily, weekly and occasional dietary guidelines to achieve a healthy and balanced diet; (2) health and gastronomy—preparation of healthy menus that include components of the MD with an impact on cardiovascular prevention and cognitive deterioration; (3) MD associated with healthy aging, hydration and macro- and micronutrients; and (4) a seminar on sugars and sweeteners—presentation of the effects of sugar consumption on health and the evaluation of different types of sugars and sweeteners and processed products and risk of diseases associated with the consumption of foods not included in the MD. All the participants attended all the sessions, with the aim of standardizing the diet of the sample, to avoid eating habits being a potential confounding factor of the results obtained as an effect of the training.

### 2.5. Outcome Measurements

#### 2.5.1. Body Composition 

Women were profiled by Level 3 International Society for the Advancement of Kinanthropometry (ISAK)-accredited anthropometrists according to ISAK guidelines [23]. The weights and heights of all participants were measured using high-quality electronic calibrated scales and a wall-mounted stadiometer, respectively. Both measurements were determined with participants wearing light clothing and no shoes. With weight in kilograms and height in centimetres, the body mass index (BMI) was calculated as weight/size^2^ (kg/m^2^). Using the World Health Organization classification, the BMI was interpreted as follows: <18.5, underweight; 18.5–24.99, normal weight; 25–29.9, overweight; and >30, obese).

A mobile anthropometer was used to determine height to the nearest millimetre (Seca 213, SECA Deutschland, Hamburg, Germany), with the participant’s head in the Frankfort Horizontal Plane position. Body perimeters were measured in triplicate (with subsequent averaging) with an anthropometric tape. Waist circumference was measured halfway between the last rib and the iliac crest by using an anthropometric tape. The hip circumference was taken horizontally in the maximum extension of the gluteus (larger posterior protrusion). With the result of both measurements, the waist-hip ratio was calculated. All circumferences included in the full ISAK profile were measured [23].

The objective of the measurements is to be able to calculate body composition based on the five-component model (fat mass, residual mass, bone mass, muscle mass and skin) proposed by Kerry Ross [24]. This model is self-evaluated because the sum of all the elements (structured weight) must be equal to the person’s actual weight. It is important to note that this model does not calculate percentage fat but percentage adiposity. Put simply, it could be said that fat is the lipid fraction contained within the adipocyte, whereas adiposity would be the lipid fraction plus the adipose cells (i.e., the lipid fraction plus water, minerals, proteins, etc.). Therefore, percentage fat is not interchangeable with percentage adiposity, the latter being 5–10% higher.

The muscle/bone index was calculated as muscle tissue divided by bone tissue in kilograms (muscle/bone). Analysis and distribution of somatotype was done through the method proposed by Heath and Carter [25].

#### 2.5.2. Body Image

The Body Shape Questionnaire, BSQ-34, is a 34-item, self-report measure of body shape and weight preoccupation initially developed to find out body image disturbance among women [26]. The questionnaire asks questions such as ‘Have you felt ashamed of your body’ and ‘Have you been so worried about your shape that you have been feeling you ought to diet’. Each item is scored from 1 to 6 (‘Never’ = 1 and ’Always’ = 6) and the total possible score is 204. Crude cut-off points have proposed that <81 correlates with no body image impairment, 81–110 with mild body image impairment, 111–140 with moderate impairment and >140 with severe impairment; nevertheless, there is no validated level between ‘normal’ and ‘abnormal’ [26], and scores were analyzed as both categorical and continuous data.

#### 2.5.3. Mediterranean Diet 

To determine the degree of adherence to the MD, a short questionnaire of 14 items was used, validated for the Spanish population and used by the MD Prevention group (Predimed) [27]. For scoring, a value of +1 was assigned to each item with a positive connotation (with regard to mean deviation, MD) and −1 for items with a negative connotation. From the sum of the values obtained for the 14 items, the degree of adherence is set, establishing two different levels: if the total score is ≥9, the diet has a satisfactory level of adherence; and if the total score is <9, the diet has a low level of adherence.

### 2.6. Statistical Analysis

Statistical analysis of the data was carried out using Jamovi 1.1.3.0. For descriptive statistics (mean ± standard deviation) and inferential analysis, the Shapiro–Wilk test was performed to determine the normality distribution. Afterwards, independent sample *t*-tests were performed to compare the different values of baseline between groups. Additionally, Levene’s test was run for equality of variances, and analysis of covariance (ANCOVA) was applied to analyze the effects of the intervention on outcomes (general linear model; time × group; BMI as covariate). Partial eta-squared (η^2^) effect sizes for time × group interaction effects were calculated. For the variables that presented significant main effects, post hoc tests (Bonferroni) were carried out. The level of significance was set at *p* ≤ 0.05. The guidelines of Cohen were followed to calculate thee effect size [28].

## 3. Results

Table 1 shows the baseline descriptive statistics, along with a comparison of baseline values between groups. The general sample is normally homogeneous. However, statistically significant differences are observed between the experimental and control groups regarding height and weight. In all cases, the experimental group presents higher values.

Table 2 gives a summary of the ANCOVA statistics. The study’s main analysis shows that there was a significant time × group difference in percentage adipose mass (*p* ≤  0.001; η^2^ = 0.654) and muscle mass (*p* ≤  0.001; η^2^ = 0.618). Post hoc analysis showed a decrease in percentage adipose mass between pre- and post-intervention in the experimental group (mean difference (MD): −2.80, *p* < 0.001, effect Size (ES): 0.471) and an increase in the control group (MD: 2.31, *p* < 0.001, ES: 0.471). There were also increases in kilograms of adipose mass (MD: 2.16, *p* < 0.001, ES: 0.357) and percentage muscle mass (MD: −2.541, *p* < 0.001, ES: 0.457) for the experimental group and decreases for the control group (MD: −1.729, *p* < 0.001, ES: 0.357 and MD = 2.035, *p* < 0.001, ES: 0.457, respectively).

In terms of kilograms of muscle mass, there were statistically significant differences only in the experimental group; there was an increase (MD: −1.97, *p* = 0.001, ES: 0.473) in muscle mass in terms of weight. However, there were also differences between the control and experimental groups at post-intervention (MD: 5.28, *p* = 0.001, ES: 1.270), with greater kilograms of muscle mass in the experimental group than the control group. About the somatotype variables, an increase in mesomorphism (MD: −0.542, *p* < 0.001, ES: 0.087) and a decrease in endomorphism (MD: 0.339, *p* = 0.040, ES: 0.0812) were observed in the experimental group. In control group, a significant decrease in mesomorphy was observed (MD: −0.254, *p* = 0.031, ES: 0.021). Nevertheless, no significant effects were found for any other variable.

## 4. Discussion

The objective of this study was to analyze the efficacy of the addition of resistance interval training in an aquatic environment to a nutritional intervention on body composition, body image perception and adherence to the MD in older women. The present study highlights different findings: the addition of resistance training in an aquatic environment to a nutritional intervention was not enough to change the perception of body image or adherence to the MD for older women. However, body composition variables were improved, in terms of loss of fat mass and gain of muscle mass.

In recent years, fat mass has been one of the most studied parameters in terms of body composition, due to its close relationship with health status. In this sense, it has been found that a greater fat mass is related to an increase in the probability of suffering cardiovascular diseases, overweight and obesity, arterial hypertension, diabetes and metabolic syndrome [29]. 

Although there are no data on body composition in women doing resistance training, there are data on body composition in women doing pilates training [29]. Comparing the results shows that, overall the women in the study of Raquel et al., 2015 [29], had lower values both before and after the intervention, whereas the level of improvement in fat percentage seems to be higher after resistance training (2.1 ± 5.75 kg), than pilates (1.04 ± 3.6 kg). No improvement occurred in the case of women in the control group, as an increase in this compartment is observed. 

For bone mass and residual mass, an increase of 0.03 ± 1.04 kg was observed in women doing pilates and 0.1 ± 1.2 kg in women doing resistance training. It appears that resistance training increases bone mineral content. This was expected, as it has been previously seen [30,31] that resistance training improves bone strength indices and functional performance in postmenopausal women. The control group in the present study was unchanged.

Muscle mass is another parameter closely related to the state of health, especially at aging and menopause stages of life, when the process of sarcopenia and other age-related muscle dysfunctions start appearing [32]. After 14 weeks of resistance training, an increase of 2 ± 5.85 kg of muscle mass was observed, while in the control group, there was a decrease of −1.2 ± 4.6 kg. If we compare with the results of Raquel et al. [29] the increase is slightly lower; 0.94 ± 4.48 kg.

In addition, coinciding with other research [33,34], it has been observed that the 14 weeks of 10–20–30 s training reduced the percentage fat mass along with an increase in percentage muscle mass. These arguments confirm the current evidence on interval training [35,36], which consists of repeated sets of high-intensity exercise interspersed with passive/active recovery because it has been shown to induce metabolic adaptations and improve body composition. In general, studies that have used exercise protocols with an intervention period of 8–24 weeks, a frequency of 2–5 times per week and a low to moderate level of exercise intensity have reported significant improvements in body composition, as indicated by significant decreases in fat mass and increases in lean mass [37]. The subjects in the present study not only lost fat mass but also increased muscle mass, which is favorable because age-related muscle mass is an important determinant of strength and physical function in older adults [38]. 

Another method proposed to estimate body composition and shape is the somatotype [39]. However, there are no studies conducted in older women that analyze this variable; there is just one that evaluated the effectiveness of the pilates method [29]. In the present study, both women in the intervention (5.79–4.87–0.59) and control (6.57–5.28–0.46) groups presented an endomorph–mesomorph somatotype in the pretest. After 14 weeks of intervention, in the intervention group, the “endomorph” component decreased and the “mesomorph” increased significantly (*p* < 0.001); (5.45–4.41–0.56). In the control group, no significant changes were observed (6.90–5.03–0.45).

As well is kown, the gold standard for measuring body composition is the Dual-energy X-ray absorptiometry (DXA), although the data are not comparable; note that the % of fat in women aged 69 ± 4 years measured by anthropometry is around 33.27 ± 4.19%, while in DXA, overweight women aged 63 ± 6 years have a % of fat mass of 38.1 ± 4.9 and women with normal weight of 31.1 ± 4.2% [40]. 

The concept of self-image (i.e., how we see ourselves) undergoes changes throughout the entire life cycle. Body image suffers modifications over the years that require adaptation and psychological accommodation [41]. The physical changes that aging entails, in a more or less gradual way, suppose a modification of the subject’s own self-image, and on many occasions, there is an abyss between the desired and the real image [41]. Studies show that approximately 50% of young women show great dissatisfaction with their physical appearance, and this is also evident in older women [42,43]. 

The relation between diet-related behaviours and body self-perception is a current theme for healthcare professionals. In a systematic review by Cristina Bouzas [44], it was noted that, generally, bodyweight satisfaction was related to having less intent to lose weight or change lifestyles. In contrast, body weight dissatisfaction was associated with a greater intent to change lifestyle or weight, a higher BMI and, specifically in women, dietary restraint. In addition, it has been reported that the body image of women can be improved only by increasing exercise, regardless of any weight change [45]. 

If the sample is classified according to score, as has been done previously in other research [46], only one participant in the intervention group was slightly preoccupied (97 pre- and 86 post-scores). In the control group, although again only one participant was slightly preoccupied, the scores were slightly higher (121 pre- and 118 post-scores). This information suggests, as previously noted [46], that the prevalence of older people suffering from body image concern is between 2.5% and 6% [46,47]. Although the differences were not significant, it has been observed that there are greater differences in the intervention group between the pre- and post-scores (52.4 ± 17.1 and 45.9 ± 12.7, respectively) than in the control group (57.4 ± 20.4 and 53.6 ± 20.9), which suggests that doing physical exercise helps to improve body image perception. 

Resistance training is widely used among older adults because physical function is closely related to strength and muscle mass, thus improving psychological well-being and health-related quality of life as well as decreasing anxiety and depression levels [48]. However, this was not observed in the present study because the perception of body image did not improve after the intervention. One of the reasons could be a lack of motivation, as it has been seen that team aerobic training or team sports training is more intrinsically motivating than resistance training, mainly due to the higher degree of social connectedness [48].

No significant differences were observed in terms of body image dissatisfaction variables, either in adherence to the MD between groups or between pre- and post-intervention. As with the Predimed score, no significant differences were observed between groups or between pre- and post-intervention. Overall, adherence to the MD was moderate in both the intervention group (5.65 ± 2.03 and 5.94 ± 2.36 pre- and post-intervention, respectively) and the control group (6.06 ± 2.14 and 5.53 ± 2.35 pre- and post-intervention, respectively). Compared to other studies, the recent scores obtained by Luigi Barrea et al. [49] and Naomi Cano-Ibáñez et al. [50] were higher.

These data suggest that because the participants did not receive an individualized nutritional program, the nutritional education they received was not sufficient to change the total Predimed score. For this type of population, individualized and specialized dietary–nutritional treatment would be recommended, with the aim of achieving greater adherence to treatment and therefore better results, as it has been noted that greater adherence to the MD is related to lower percentages of fat mass and higher BMI values in this population [51]. 

The present study has some limitations. Firstly, the study included only female patients. Because of the gender-specific response, our results may not be generalizable to all elderly populations. The levels of daily PA were not assessed using self-reported questionnaires such as the International Physical Activity Questionnaire or using measurement devices such as accelerometers or smart watches. In addition, the method of measuring body composition must be considered, since anthropometry was used and not DXA, which is considered the gold standard for body composition assessments. Finally, diet control or nutritional supplementation during the intervention was not analyzed; there could also be an association with body composition. Food quality was assessed, but it was not feasible to evaluate the quantity. 

Future research should consider the limitations presented above. Researchers in the field are asked to evaluate more specific information on the amount of food and supplements ingested by the participants. In addition to assessing total daily PA, the activity bracelets should consider the training sessions they performed in the intervention.

## 5. Conclusions

The addition of resistance training in an aquatic environment to a nutritional intervention was not sufficient to change the perception of body image and adherence to the MD in older women. However, it does produce an improvement in body composition, through the increase of muscle mass and decrease of fat mass. To improve eating habits and body image perception, specific intervention and individualized treatment is necessary for this population.

## Figures and Tables

**Table 1:** Baseline characteristics of study participants.

Variables	Intervention Group (*n* = 17)			Control Group (*n* = 17)					
	Baseline			Baseline			Baseline Differences		
	Mean		SD	Mean		SD	*t*	*p*	ES
Age (Years)	69.6	±	5.0	67.7	±	3.6	1.257	0.218	0.431
Height (cm)	162.0	±	7.9	154.0	±	5.4	3.347	0.002	1.148
Weight (kg)	75.3	±	12.8	66.9	±	10.2	2.122	0.042	0.728
BMI (kg/m^2^)	28.8	±	4.7	28.2	±	4.2	0.385	0.703	0.132

BSQ = Body Shape Questionnaire; SD = Standard deviation; *t* = *t* value; *p* = *p* value; ES = Effect size.

**Table 2:** Comparison of characteristics at baseline and post-intervention (ANCOVA).

Variables	Intervention Group (*n* = 17)						Control Group (*n* = 17)						Effect Time			Effect Time × Group		
	Baseline			Post			Baseline											
	Mean		SD	Mean		SD	Mean		SD	Mean		SD	*F*	*p*	η^2^*p*	*F*	*p*	η^2^*p*
Body Composition																		
Weight (kg)	75.3	±	12.8	75.3	±	13.2	66.9	±	10.2	67.4	±	10.3	1.065	0.310	0.033	0.329	0.570	0.011
% fat mass	32.3	±	4.5 *	29.5	±	3.9 *	34.2	±	4.1 *	36.5	±	3.9 *	0.205	0.654	0.007	58.649	<0.001	0.654
% residual mass	11.8	±	2.5	12.1	±	2.4	10.6	±	1.4	10.3	±	1.2	0.319	0.577	0.010	0.434	0.515	0.014
% muscle mass	40.5	±	3.4 *	43.0	±	2.4 *	41.6	±	2.8 *	39.6	±	2.7 *	1.160	0.291	0.036	50.09	<0.001	0.618
% bone mass	10.2	±	1.5	10.2	±	1.5	8.5	±	0.9	8.5	±	0.9	2.164	0.151	0.065	0.194	0.662	0.006
% skin	5.1	±	0.7 ^#^	5.15	±	0.8 ^#^	5.0	±	0.4 ^#^	5.1	±	0.5 ^#^	4.526	0.041	0.127	3.827	0.060	0.110
kg fat mass	24.5	±	6.2 *	22.4	±	5.3 *	22.9	±	4.3 *	24.7	±	4.7 *	1.110	0.300	0.035	59.27	<0.001	0.657
kg muscle mass	30.5	±	5.6 *	32.5	±	6.1 *	27.8	±	4.7	26.6	±	4.5	0.140	0.710	0.005	22.118	<0.001	0.416
kg residual mass	8.9	±	2.3	9.1	±	2.1	7.2	±	1.8	6.9	±	1.6	1.330	0.258	0.041	2.56	0.120	0.076
kg bone mass	7.6	±	1.2	7.7	±	1.2	5.7	±	0.9	5.7	±	0.9	3.218	0.083	0.094	0.441	0.511	0.014
kg skin	3.8	±	0.6	3.8	±	0.6	3.3	±	0.3	3.4	±	0.3	4.120	0.051	0.117	0.202	0.656	0.006
WHR	0.9	±	0.1	0.9	±	0.1	0.9	±	0.1	0.9	±	0.1	0.297	0.590	0.009	4.377	0.055	0.124
Endomorph	5.79	±	1.72 *	5.45	±	1.67 *	6.57	±	1.22	6.90	±	1.50	0.000815	0.977	0.000	15.0	<0.001	0.011
Mesomorph	4.87	±	1.26 *	5.41	±	1.41 *	5.28	±	1.56 *	5.03	±	1.40 *	5.65	0.023	0.003	43.01	<0.001	0.021
Ectomorph	0.59	±	0.69	0.56	±	0.60	0.46	±	0.47	0.45	±	0.47	0.580	0.451	0.017	0.183	0.672	0.005
Body Image																		
BSQ	52.4	±	17.1	45.9	±	12.7	57.4	±	20.4	53.6	±	20.9	2.050	0.162	0.062	0.482	0.493	0.015
Mediterranean Diet																		
Predimed	5.7	±	2.0	5.9	±	2.36	6.1	±	2.1	5.5	±	2.3	0.198	0.659	0.006	3.128	0.087	0.092

BMI = Body Mass Index; kg = kilograms; WHR: waist-hip ratio; SD = Standard deviation; *t* = *t* value; *p* = *p* value; ES = Effect size; Mean differences are considered significant when *p* < 0.05; ^#^ differences in time; * differences in time × group.

## Data Availability

The data presented in this study are available on request from the corresponding author. The data are not publicly available due to the fact that they consist in personal health information.
